# Length Estimation of Pneumatic Artificial Muscle with Optical Fiber Sensor Using Machine Learning

**DOI:** 10.3390/s25072221

**Published:** 2025-04-01

**Authors:** Yilei Ni, Shuichi Wakimoto, Weihang Tian, Yuichiro Toda, Takefumi Kanda, Daisuke Yamaguchi

**Affiliations:** Graduate School of Environmental, Life, Natural Science and Technology, Okayama University, Okayama 700-8530, Japan; ni22@s.okayama-u.ac.jp (Y.N.); den_19@s.okayama-u.ac.jp (W.T.); ytoda@okayama-u.ac.jp (Y.T.); kanda-t@okayama-u.ac.jp (T.K.); yamaguchi20@okayama-u.ac.jp (D.Y.)

**Keywords:** McKibben artificial muscle, machine learning, optical fiber, motion estimation

## Abstract

A McKibben artificial muscle is a soft actuator driven by air pressure, characterized by its flexibility, lightweight design, and high power-to-weight ratio. We have developed a smart artificial muscle that is capable of sensing its motion. To enable this sensing function, an optical fiber was integrated into the sleeve consisting of multiple fibers and serving as a component of the McKibben artificial muscle. By measuring the macrobending loss of the optical fiber, the length of the smart artificial muscle is expected to be estimated. However, experimental results indicated that the sensor’s characteristics depend not only on the length but also on the load and the applied air pressure. This dependency arises because the stress applied to the optical fiber increases, causing microbending loss. In this study, we employed a machine learning model, primarily composed of Long Short-Term Memory (LSTM) neural networks, to estimate the length of the smart artificial muscle. The experimental results demonstrate that the length estimation obtained through machine learning exhibits a smaller error. This suggests that machine learning is a feasible approach to enhancing the length measurement accuracy of the smart artificial muscle.

## 1. Introduction

The McKibben artificial muscle [1,2], a typical pneumatic soft actuator, contracts axially and expands radially, with its contraction displacement serving as the mechanical output. This actuator has a simple structure, consisting of a rubber tube and sleeve fibers, and is known for its excellent compatibility with human applications and high power-to-weight ratio. In recent years, McKibben artificial muscles have been incorporated into general-purpose for power-assist and rehabilitation devices, which are now commercially available [3,4].

Despite its effectiveness as an actuator, the McKibben artificial muscle faces challenges in control due to the mechanical nonlinearity of its rubber materials and the anisotropy of the generated force, which results from friction between the rubber and the fibers. These factors make it more difficult to accurately sense the state of artificial muscles. As a result, additional sensors, such as laser displacement sensors and linear encoders, are often required for precise measurement. However, the integration of these additional sensors frequently leads to increased mechanical rigidity and system complexity. Many previous studies have attempted to overcome these challenges by using modeling and machine learning methods to achieve feedforward control of sensorless artificial muscles [5,6]. These approaches rely on pre-optimized parameters and predictive models, eliminating the need for onboard sensors. However, when feedback control is required, additional sensors are typically necessary for accurate measurement.

Optical fibers can function as flexible fiber sensors, and their flexibility makes them well-suited for sensing soft actuators. They have been used to measure the curvature and displacement of fluid pressure-driven soft actuators [7,8,9,10,11]. In our previous study, we developed an artificial muscle capable of estimating its displacement by incorporating optical fibers into the sleeve of a McKibben artificial muscle [12]. Since McKibben artificial muscles inherently have fiber sleeves, no additional processing was required to attach the optical fibers. Moreover, the optical fibers serve not only as a sensing element but also as an actuation component. We call it “smart artificial muscle” in this paper. Optical fibers exhibit a property known as macrobending loss, where the amount of light propagating through the fiber decreases as the radius of curvature decreases. These fibers are flexible and lightweight, allowing for the simultaneous integration of the fabrication processes of both artificial muscles and sensors. When air pressure is applied to artificial muscle, the muscle expands circumferentially and contracts axially, decreasing the radius of curvature of the integrated optical fiber. By measuring the amount of light propagating through the optical fiber, the muscle’s self-displacement can be estimated. In this previous research, a smart artificial muscle was designed for low air pressure driving by very soft rubber, enabling operation at pressures below 100 kPa. The optical fiber used had a diameter of 0.5 mm, which was relatively thick to prevent microbending loss, as described in Section 2.2. Under these design limitations, the muscle’s displacement was successfully estimated using the optical fiber sensor. The sensor output exhibited a linear relationship with the muscle’s contraction displacement. This pressure range is lower than that typically required to drive general pneumatic artificial muscles, which use standard rubber tubes. Moreover, the diameter of the optical fiber must be selected carefully to avoid being excessively small. We also fabricated a smart artificial muscle with a standard hardness rubber tube and a thinner optical fiber. When pressure was applied, the sensor output depended not only on the contraction displacement but also on the applied pressure and external load.

On the other hand, in recent years, with the advancement of artificial intelligence research, machine learning has been applied to realize sensing functions for soft actuators and to improve their accuracy [13,14,15,16,17,18,19,20]. Some studies apply LSTM (Long Short-Term Memory) networks, which is a powerful recurrent neural network designed to overcome exploding and vanishing gradient issues that typically arise when learning long-term dependencies, even with extended time lags, to enhance soft robot perception and control using embedded sensors for real-time kinematic and force estimation, dynamic response prediction, and actuator calibration, while improving flexibility and precision without rigid sensors [15,16,17,18,19,20].

In this study, we propose a method to enhance the accuracy of length estimation in smart artificial muscles using thinner optical fibers driven by general air pressure levels. This method utilizes the LSTM. We explored a technique for estimating the length of a smart artificial muscle by sensing the applied air pressure and the variation in the light propagating through the optical fiber. This information was used as time-series data for training the neural networks. As a result, this study achieved high displacement estimation accuracy under less restrictive design conditions for the smart artificial muscle.

## 2. Materials

### 2.1. McKibben Artificial Muscle

**Figure 1:**
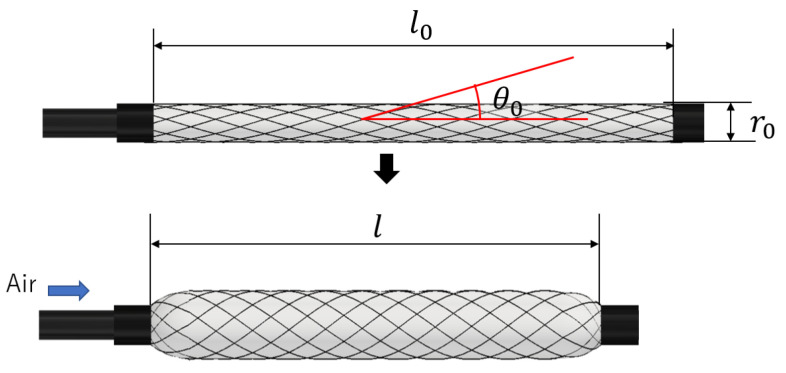
Configuration of McKibben artificial muscle.

The McKibben actuator consists of a rubber tube and a sleeve of fibers covering the rubber tube. It is lightweight, flexible, structurally simple, and possesses a high force-to-weight ratio. A model of a McKibben artificial muscle is shown in Figure 1. When air is injected and pressure is applied to the rubber tube, the sleeve fibers act as a pantograph mechanism, causing the artificial muscle to expand radially and contract axially. The resulting contractile force and displacement are typically used as mechanical outputs in robotic mechanisms.

Focusing on the shape of the fibers in the sleeve, the spiral shape changes with axial contraction and radial expansion. The radius of curvature of the spiral fiber can be expressed using Equation (1), which is a modified version from the previous study [12].

*R* represents the radius of curvature of the fiber, *r*_0_, *l*_0_ and $\theta_{0}$ are the initial diameter, initial length, and initial braided angle of the fibers, respectively. *l* represents the length of the artificial muscle, when the air pressure is applied. The parameters *r*_0_, *l*_0_ and $\theta_{0}$ are constant values determined during the fabrication of the artificial muscle, and *R* varies with *l*.

(1) $$R=\frac{r_{0}}{sin\theta_{0}\sqrt{1-{\left(\frac{l}{l_{0}}\right)}^{2}{cos}^{2}\theta_{0}}}$$

### 2.2. Optical Fiber

**Figure 2:**
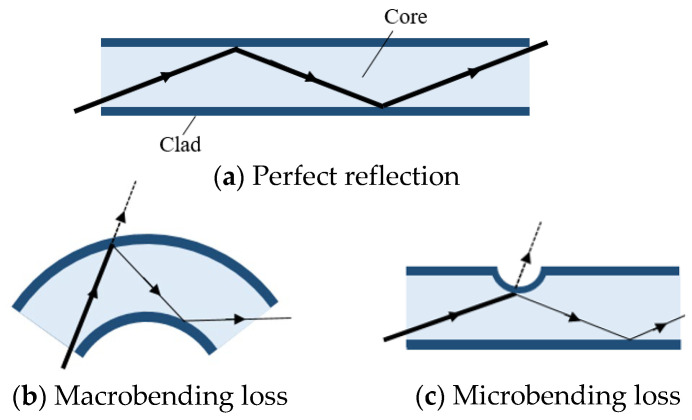
Propagated light in optical fiber, (**a**) is a perfect reflection, (**b**) is macrobending loss, and (**c**) is microbending loss.

The structure of an optical fiber is shown in Figure 2a. An optical fiber consists of two layers: a central core and a surrounding cladding with a lower refractive index than the core. When the angle of light incidence exceeds the critical angle, light is confined within the core and propagates through the fiber via total internal reflection.

However, when the fiber is bent, as illustrated in Figure 2b, the incidence angle decreases below the critical angle, causing some light to leak from the fiber—a phenomenon known as macrobending loss [21,22]. This property leads to a reduction in the amount of light propagating through the fiber as the radius of curvature decreases.

Moreover, as depicted in Figure 2c, when the surface of the optical fiber is partially loaded and surface deformation becomes locally significant, some light leaks out at the deformation point. This phenomenon is referred to as microbending loss [23].

The total amount of light propagating through the optical fiber is determined by both macrobending loss and microbending loss.

### 2.3. Configuration of Smart Artificial Muscle

**Figure 3:**
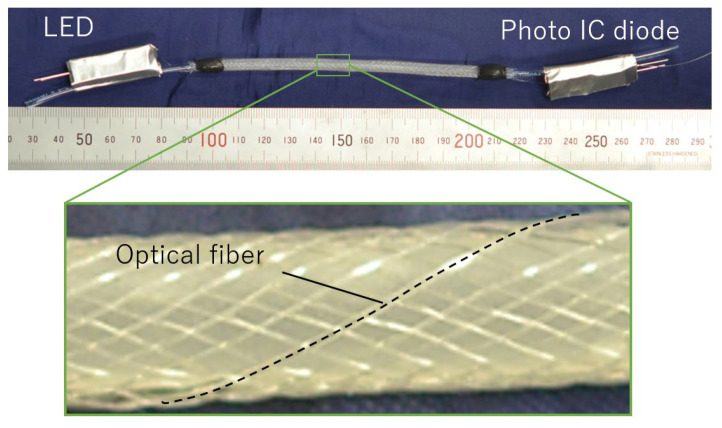
Fabricated smart artificial muscle with optical fiber.

Figure 3 shows the smart artificial muscle incorporating an optical fiber with a diameter of 0.25 mm. The easy fabrication process of this structure using a braider machine was established in the previous report [12]. The actuation part’s length and diameter are 100 mm and 5 mm. A total of 32 fibers (one optical fiber and 31 general synthetic fibers) are braided together.

One of the sleeve fibers is an optical fiber. To function as a sensor, the optical fiber requires a light emitter at one end and a light receiver at the other to measure the transmitted light intensity.

The artificial muscle has an LED (C503B-GAN-CB0F0791, Cree LED, Durham, NC, USA) at one end for emitting light into the optical fiber and a Photo IC diode (S13948-01SB, Hamamatsu Photonics K.K., Hamamatsu, Japan) at the other end for receiving the propagated light. The LED and photo IC diode were installed in 3D-printed connectors, and covered with an aluminum sheet to shield them from external light interference.

### 2.4. Fundamental Characteristics of Smart Artificial Muscle

**Figure 4:**
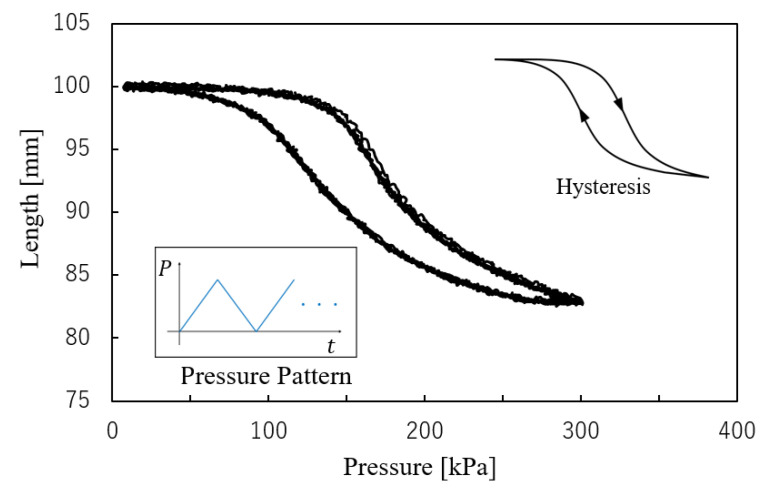
Relation between pressure and length of smart artificial muscle.

For checking the actuation ability, air pressure was applied from 0 kPa to 300 kPa over 30 s, followed by a reduction to 0 kPa over the next 30 s. This cycle was repeated five times, with the length of the smart artificial muscle and applied pressure measured throughout. The relationship between these variables is shown in Figure 4.

The maximum air pressure of 300 kPa is a standard level for McKibben artificial muscles. In its most contracted state, the length of the artificial muscle is 83 mm (initial length: 100 mm). Hysteresis was observed, which is a well-known characteristic of this type of soft actuator due to its inherent mechanical properties.

**Figure 5:**
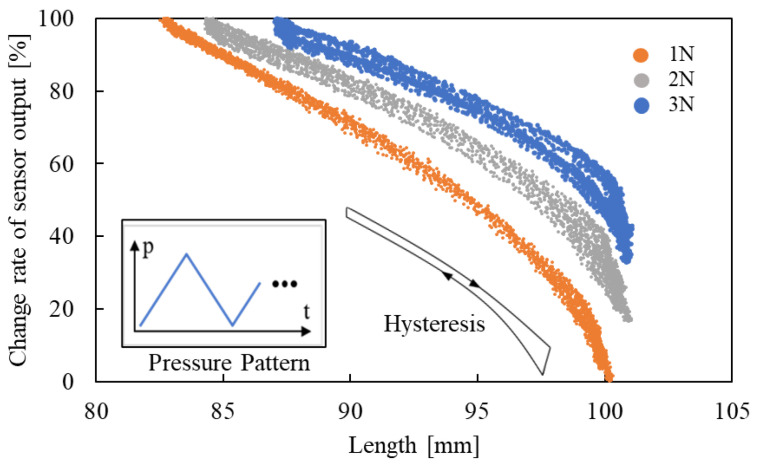
Relation between length and change rate of sensor output with loads.

In the fundamental experiments, the length of the smart artificial muscle and the change rates of sensor output were measured while the muscle was set vertically. Figure 5 presents the measurement results for loads of 1.0 N, 2.0 N, and 3.0 N, which were suspended from the smart artificial muscle to counteract its contraction force.

Focusing on the result under each load condition, the sensor output changes as the length changes. The primary reason for this phenomenon is macrobending loss. The radius of curvature *R* of the optical fiber which is one of the sleeve fibers varies with the length *l*, as explained in Equation (1). It leads macrobending loss.

Additionally, the load affects the sensor output because the sleeve is subjected to tensile stress from the loads, resulting in high-stress points where fibers, including the optical fiber, are compressed at contact points. This compression causes microbending loss. From Figure 5, hysteresis and nonlinearity are observed.

**Figure 6:**
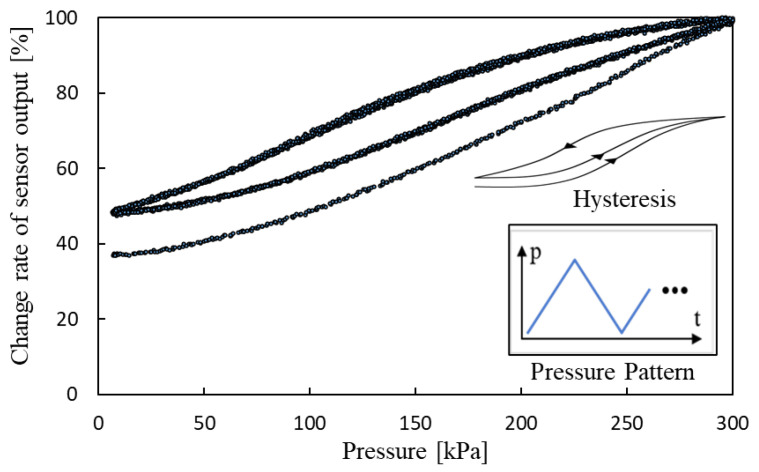
Relation between pressure and change rate of sensor output (length of smart artificial muscle is held in initial length).

Moreover, with the conditions of the length held at the initial length by securing both ends of the smart artificial muscle, the air pressure was changed, and the sensor output was measured. Figure 6 shows the result, the sensor output varied with increasing air pressure, even when the length was constant. This is because the fibers are stressed by the pressurized rubber, and the optical fiber is pressed at the points where the optical fiber is sandwiched between the other fibers and the pressurized rubber. This is also the cause of microbending loss. This observation indicates the high hysteresis and nonlinearity of sensor output. The first hysteresis loop differs from the subsequent loop, suggesting dependency on time and past conditions.

These phenomena, nonlinearity, hysteresis, and time/past condition dependency, stem from the intrinsic properties of polymer soft materials. These characteristics are generally observed in soft materials, and the main body of the smart artificial muscle is entirely composed of soft materials.

In summary, the sensor output is influenced by length, loads, and air pressure, and it exhibits nonlinearity, hysteresis, and dependency on time/past conditions.

### 2.5. Structure of Machine Learning

In this section, we describe the process of estimating the length of the smart artificial muscle using neural networks. Since estimating the length of the smart artificial muscle is a time-series task, we utilized a powerful recurrent neural network, Long Short-Term Memory (LSTM) [24], to perform this task. This method provides a more accurate estimation of the muscle’s length compared to traditional linear regression, particularly when the muscle sensor value is influenced by microbending loss.

**Figure 7:**
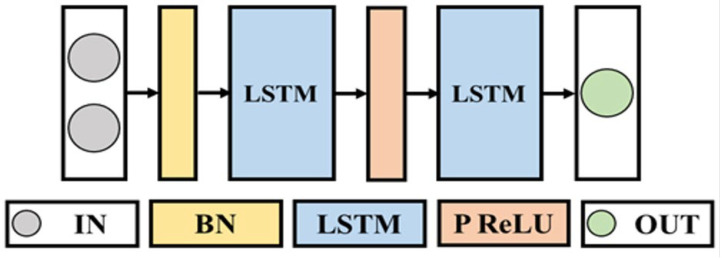
Structure of neural networks.

As shown in Figure 7, the neural network consists of three LSTM layers, a Batch Normalization (BN) layer, a Parametric Rectified Linear Unit (PReLU) activation function layer, and a fully connected layer.

The input data to the model consists of two parts:

Applied pressure value: measured using a pressure sensor attached to the artificial muscle.

Fiber optical sensor values: obtained from the optical fiber embedded in the sleeve of the artificial muscle, which reflects the loss due to deformation for both macrobending loss and microbending loss.

Since the LSTM model requires continuous data processing, we applied a lookback window mechanism to provide historical context for each prediction. Depending on the hyperparameter settings, the lookback value is set to 30 time steps with return sequences to ensure that the model has access to past sensor readings when estimating the current muscle length. This approach allows the model to account for time dependence and improves the stability of the predictions.

The output of the model is the estimated length of the artificial muscle and is compared to the actual displacement values obtained through the laser displacement sensor for evaluation. To improve the reliability of the data, the sensor readings were preprocessed, a moving average filter was applied to smooth out noise fluctuations, and the values were normalized for stable model training.

The LSTM unit comprises a cell, an input gate, an output gate, and a forget gate, which together allow the network to regulate its state over time. The cell stores values over arbitrary time intervals, while the three gates regulate the flow of information in and out of the cell. The LSTM architecture is composed of recurrently connected subnetworks, known as memory blocks. These blocks maintain their state over time and regulate information flow through nonlinear gating units.

**Figure 8:**
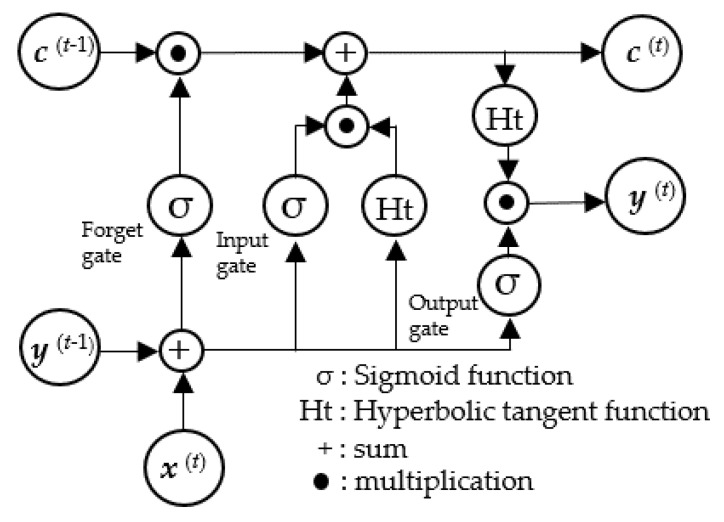
Architecture of LSTM block.

Figure 8 illustrates the architecture of the LSTM block, showing the gates, input signal ***x***^(*t*)^, output ***y***^(*t*)^, cell value ***c***^(*t*)^, and activation functions (sigmoid function and hyperbolic tangent function), and the output of the block is recurrently connected back to the block input and all gates [24].

Batch Normalization (BN) plays a crucial role in the training process by normalizing the intermediate layer activations, making the training process more stable and the gradients more consistent. This leads to gradients that are more stable and less prone to exploding or vanishing, enabling faster and more effective optimization, and improving robustness to hyperparameter settings [25,26,27].

Parametric Rectified Linear Unit (PReLU), first proposed by Kaiming He in 2015, addresses the limitations of the standard ReLU activation function, particularly the issue known as “dying ReLU”, where neurons become inactive when the input is negative. To prevent this, PReLU introduces a small negative slope α, allowing gradients to pass through even when x is negative. This slope is a learnable parameter; when it is fixed, the function is referred to as Leaky ReLU. The PReLU algorithm converges faster, has lower training errors, and does not lead to overfitting despite the introduction of the α parameter [28].

Hyperparameters were set as shown in Table 1.

### 2.6. Experimental Setup

**Figure 9:**
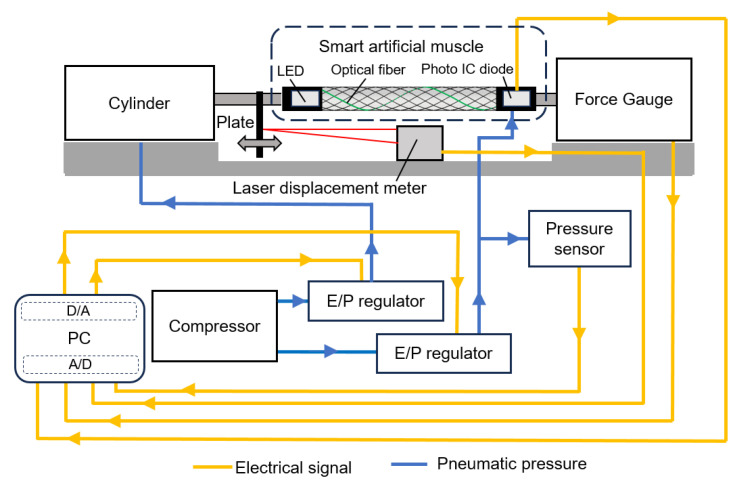
Experimental system.

Figure 9 shows the experimental system which was used to collect machine learning data. One side of the smart artificial muscle is fixed to the air cylinder, while the other side is fixed to the force gauge. By applying the air pressure to the air cylinder, which is controlled by a PC via an electric/pneumatic (E/P) regulator, the arbitrary load can be added to the smart artificial muscle, and the force can be observed by the force gauge.

Additionally, the air pressure to the smart artificial muscle can be independently controlled by the PC and the E/P regulator. Because air has viscosity and compressibility, the actual pressure value in the smart artificial muscle differs from the commanded value from the PC. Therefore, a pressure sensor is installed to measure the actual pressure value in the smart artificial muscle.

A flat plate is arranged at one end of the smart artificial muscle. This plate moves together with the smart artificial muscle, and the displacement is measured by a laser displacement meter, allowing the length of the smart artificial muscle to be observed.

The air pressure value in the smart artificial muscle (from the pressure sensor), force value (from the force gauge), actual length of the smart artificial muscle (from the laser displacement meter), and sensor output of the optical fiber (from the photo IC diode) are collected by the PC.

## 3. Results and Discussion

### 3.1. Test Results

Air pressure is applied to the smart artificial muscle while an arbitrary load is exerted by the cylinder. The sampling frequency was set to 10 Hz, with four load settings (1.2 N, 1.7 N, 2.1 N, and 2.6 N) and three driving patterns described below, resulting in 12 combinations. To prevent the artificial muscles from being damaged, the maximum load of the artificial muscles in the experiments of this paper is 3 N.

[Step pattern]: The applied pressure starts from 0 and increases in steps of 50 kPa, reaching 300 kPa, with each step lasting five seconds.

[Triangle wave pattern]: The air pressure increases from 0 kPa to 300 kPa over 30 s at a steady rate, then decreases back to 0 kPa over the next 30 s.

[Random pattern]: Air pressure randomly changes between 10 kPa and 300 kPa every four seconds.

One minute of data from each combination, a total of 12 min of training data collected across different load conditions (1.2 N, 1.7 N, 2.1 N, and 2.6 N), was used as the training dataset, and the machine learning, as described in Figure 7, was conducted with hyperparameters shown in Table 1.

To evaluate the machine learning contribution clearly, we introduced a multiple linear regression model, expressed by the following equation, and compared the results:

(2) $$l_{est}=as+bp+c$$

Here, *l_est_* represents the estimated length of the artificial muscle, *s* is the optical fiber sensor output, and p is the applied pressure when driving the smart artificial muscle in practical use, the sensor value and applied pressure are known. The regression system’s coefficients, *a* and *b*, and constant term *c* were calculated using the same datasets employed for the machine learning model to ensure a fair comparison.

As a comparative model to the machine learning approach, we introduced a Multilayer Perceptron (MLP) model. We replaced the LSTM layers in the previously described LSTM model with fully connected layers, each utilizing a hyperbolic tangent activation function. Furthermore, the lookback window in the input data was removed, while the same training data and hyperparameters were used, except for the removal of the lookback window. This created an MLP model. Subsequently, we compared the predictive performance of the three models to assess their differences in performance.

For validating the LSTM model, the length of the smart artificial muscle was estimated by three pressure patterns and four load patterns mentioned above.

**Figure 10:**
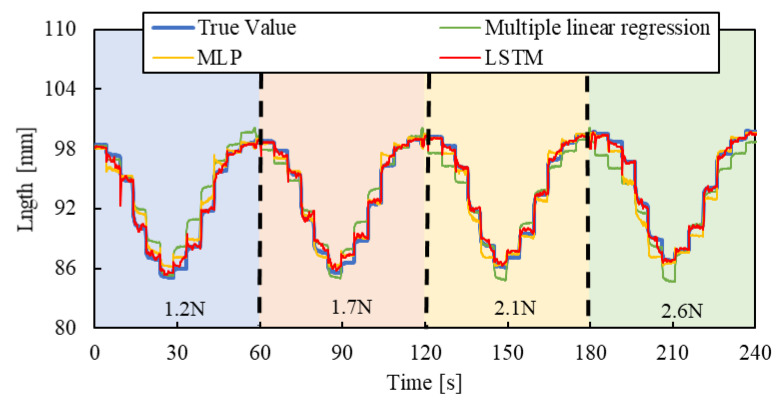
Length estimation for Step pattern. The blue, red, yellow, and green background sections correspond to loads of 1.2 N, 1.7 N, 2.1 N, and 2.6 N.

**Figure 11:**
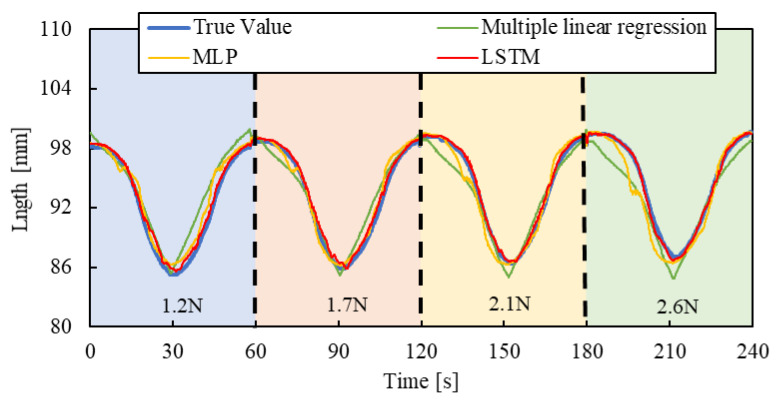
Length estimation for Triangle wave pattern. The blue, red, yellow, and green background sections correspond to loads of 1.2 N, 1.7 N, 2.1 N, and 2.6 N.

**Figure 12:**
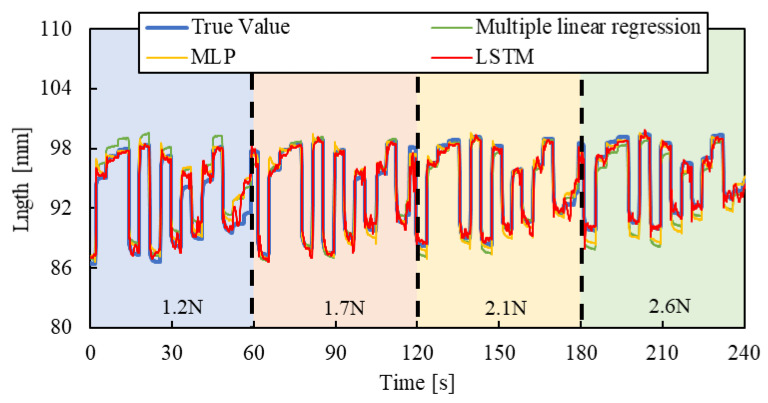
Length estimation for Random pattern. The blue, red, yellow, and green background sections correspond to loads of 1.2 N, 1.7 N, 2.1 N, and 2.6 N.

Figure 10, Figure 11 and Figure 12 show the results. Each result indicates that the LSTM model can estimate the length of the smart artificial muscle more accurately compared to the multiple linear regression model. While the error in the multiple linear regression model becomes large under some load conditions, the error in the LSTM model remains consistently low across all load cases when compared to the multiple linear regression results. In addition, compared to the MLP model, the LSTM model exhibits a smaller error in the all patterns.

We quantified the performance by calculating the Root Mean Square Error (RMSE) between the estimated value and the actual value using the following Equation (3):

(3) $$RMSE=\sqrt{\frac{\sum_{i=1}^{K}{\left({\tilde{y}}_{i}-y_{i}\right)}^{2}}{K}}$$

where K is the total number of data points, ${\tilde{y}}_{i}$ is the estimated value, and $y_{i}$ is the actual value. The experimental results are shown in Table 2. For the step and triangle wave inputs, the RMSE of the LSTM model is approximately 0.4 mm, while the RMSE of the multiple linear regression model and MLP are about 1.3 mm and 0.8 mm, 1.4 mm and 1.0 mm, respectively. For random step inputs, the RMSE of the machine learning model increases to about 0.8 mm, but it still outperforms the multiple linear regression model and MLP.

### 3.2. Validation of Generality

To evaluate the model’s generalization ability, the load of 2.4 N, 1.9 N, and 1.4 N were newly set by the air cylinder, and the length of the smart artificial muscle was estimated for step, triangle wave, and random inputs by the mechanical learning model.

**Figure 13:**
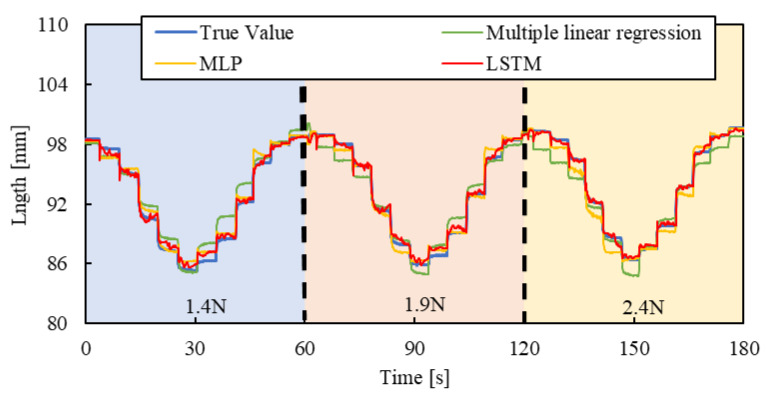
Length estimation for Step pattern. The blue, red, and yellow background sections correspond to loads of 1.4 N, 1.9 N, and 2.4 N.

**Figure 14:**
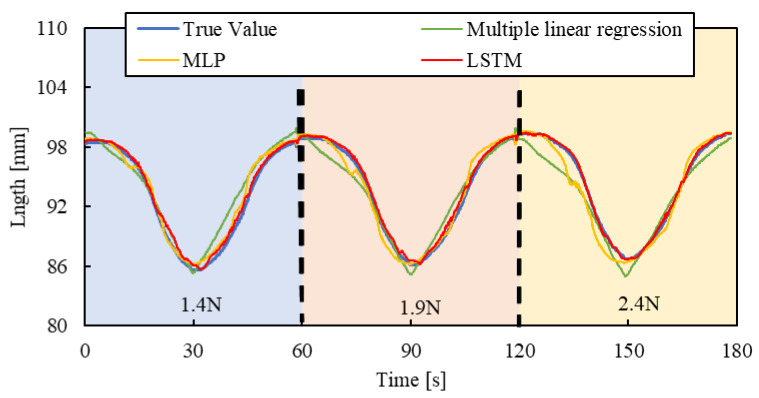
Length estimation for Triangle wave pattern. The blue, red, and yellow background sections correspond to loads of 1.4 N, 1.9 N, and 2.4 N.

**Figure 15:**
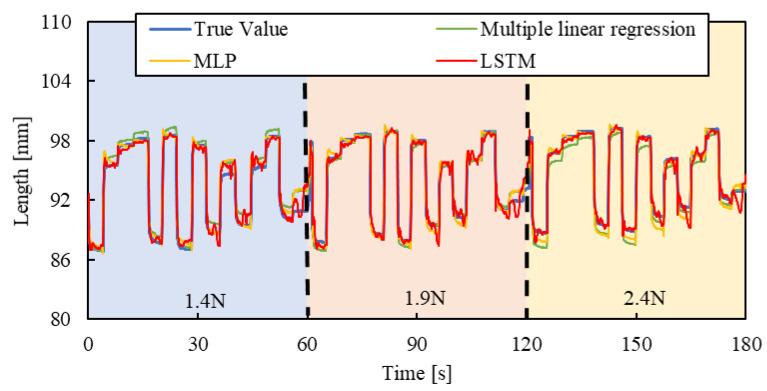
Length estimation for Random pattern. The blue, red, and yellow background sections correspond to loads of 1.4 N, 1.9 N, and 2.4 N.

Figure 13, Figure 14 and Figure 15 show the results. Results of a multiple linear regression model are also represented together. We can recognize that even with the new load conditions, which are not included in establishing the machine learning model, the length of the smart artificial muscle is estimated correctly. In addition, compared to the results of the MLP model and multiple linear regression model, those of the LSTM model are superior.

RSMEs were calculated using Equation (3) for Figure 13, Figure 14 and Figure 15, and were compared to those for Figure 10, Figure 11 and Figure 12. The RSME values are almost identical in each case. The experimental results are shown in Table 3. This indicates that the model performs well not only on the load modes included in the training dataset but also on load settings not explicitly included during training.

Moreover, a manual air pressure regulator was used to verify the model’s ability which should be able to accurately estimate the length of the smart artificial muscle under completely random pressure conditions. This experiment was conducted under a load of 1.5 N. This air pressure regulator adjusts the air pressure by turning a rotational lever by the hand. We applied random pressure to the smart artificial muscle by arbitrarily turning the lever. The length of the smart artificial muscle was then estimated. This experiment was conducted under complex and practical conditions.

**Figure 16:**
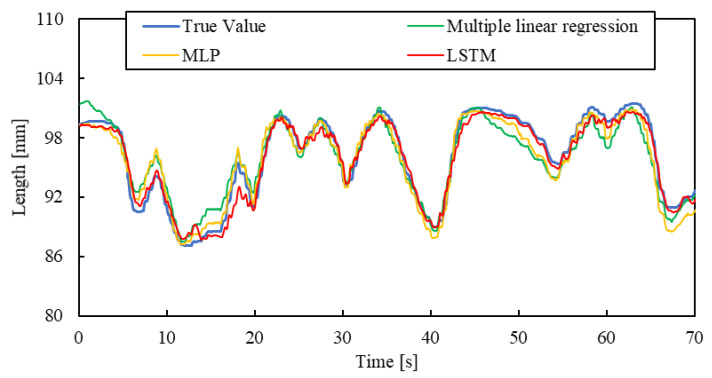
Length estimation for arbitrary motion by manual air regulator.

The result is shown in Figure 16. The estimated length of the smart artificial muscle by the machine learning successfully aligned with the actual length value. When comparing the result by the multiple linear regression, the accuracy of it is higher. The RSMEs of the LSTM, MLP, and multiple linear regression are 0.80 mm, 1.13 mm, and 1.41 mm, respectively. These results show that machine learning models, especially LSTM models, work well even under complex and practical conditions.

## 4. Conclusions

This study has demonstrated the effectiveness of a machine learning model using a Long Short-Term Memory (LSTM) neural network for estimating the length of an optical fiber-type smart artificial muscle. The experiments showed that the model can generalize effectively to new load conditions and arbitrary air pressure conditions using a manual air regulator. The RMSE of the estimated length using the machine learning model was consistently smaller than that of the multiple linear regression model in all experimental cases. In a previous study where machine learning was not used, the design of the smart artificial muscle faced limitations. Specifically, the thickness of the optical fiber and the hardness of the rubber tube should be carefully selected to avoid microbending loss. In this study, we found that machine learning can effectively address the problems caused by microbending loss. This result suggests that smart artificial muscles with optical fiber sensors can now be designed without the need for meticulous material selection. In future work, we plan to implement feedback control for the smart artificial muscle based on the length estimation model using machine learning. Specifically, our goal is to integrate this length estimation into feedback control systems, such as PID control, to enhance the actuation performance of the artificial muscle. This will enable precise control of the smart artificial muscle and facilitate its application as an actuator for soft robots. Since we have not yet quantitatively measured the repeatability and durability of the optical fiber and smart artificial muscle, these measurements will be addressed in future work.

## Figures and Tables

**Table 1:** Hyperparameters for Machine Learning.

Parameter	Setting Value and Name
LSTM layers	Left block:1, Right block:2 (Figure 7)
LSTM layer neurons	150
Epochs	1000
Batch size	256
Data split rate	0.9
Lookback	30
Learning rate	0.0001
Optimization algorithm	Adam
Loss function	Mean squared Error

**Table 2:** RSME of machine learning model and multiple linear regression model.

	RMSE [mm]			
Model	Step	Triangle Wave	Random	
LSTM	0.43	0.41	0.83	
MLP	0.82	1.04	0.93	
Multiple linear regression	1.29	1.43	1.04	

**Table 3:** RSME of machine learning model between Figure 10, Figure 11 and Figure 12, and Figure 13, Figure 14 and Figure 15 for confirming generalization ability.

	RMSE [mm]		
Machine Learning Data	Step	Triangle Wave	Random
Figure 10, Figure 11 and Figure 12	0.43	0.41	0.83
Figure 13, Figure 14 and Figure 15	0.41	0.38	0.75

## Data Availability

Data are contained within the article.
